# Supplementary material for: Structure and function of Semaphorin-5A glycosaminoglycan interactions
Source: Nat Commun. 2024 Mar 28;15:2723. doi: 10.1038/s41467-024-46725-7 (PMC10978931; doi:10.1038/s41467-024-46725-7)
Supplement: Supplementary file 1 — Supplementary Information [file 41467_2024_46725_MOESM1_ESM.pdf]

Supplementary Information for

Structure and function of Semaphorin-5A glycosaminoglycan interactions

Gergely N. Nagy*, Xiao-Feng Zhao, Richard Karlsson, Karen Wang, Ramona Duman, Karl Harlos, Kamel El Omari, Armin Wagner, Henrik Clausen, Rebecca L Miller*, Roman J. Giger*, E. Yvonne Jones*

*Corresponding authors. Emails: nagy.gergely.nandor@vbk.bme.hu (G.N.N.), rmiller@sund.ku.dk (R.L.M.), rgiger@med.umich.edu (R.J.G.), yvonne.jones@strubi.ox.ac.uk (E.Y. J).

The file includes:

Supplementary Figures 1 to 14
Supplementary Tables 1 to 2
Supplementary References.

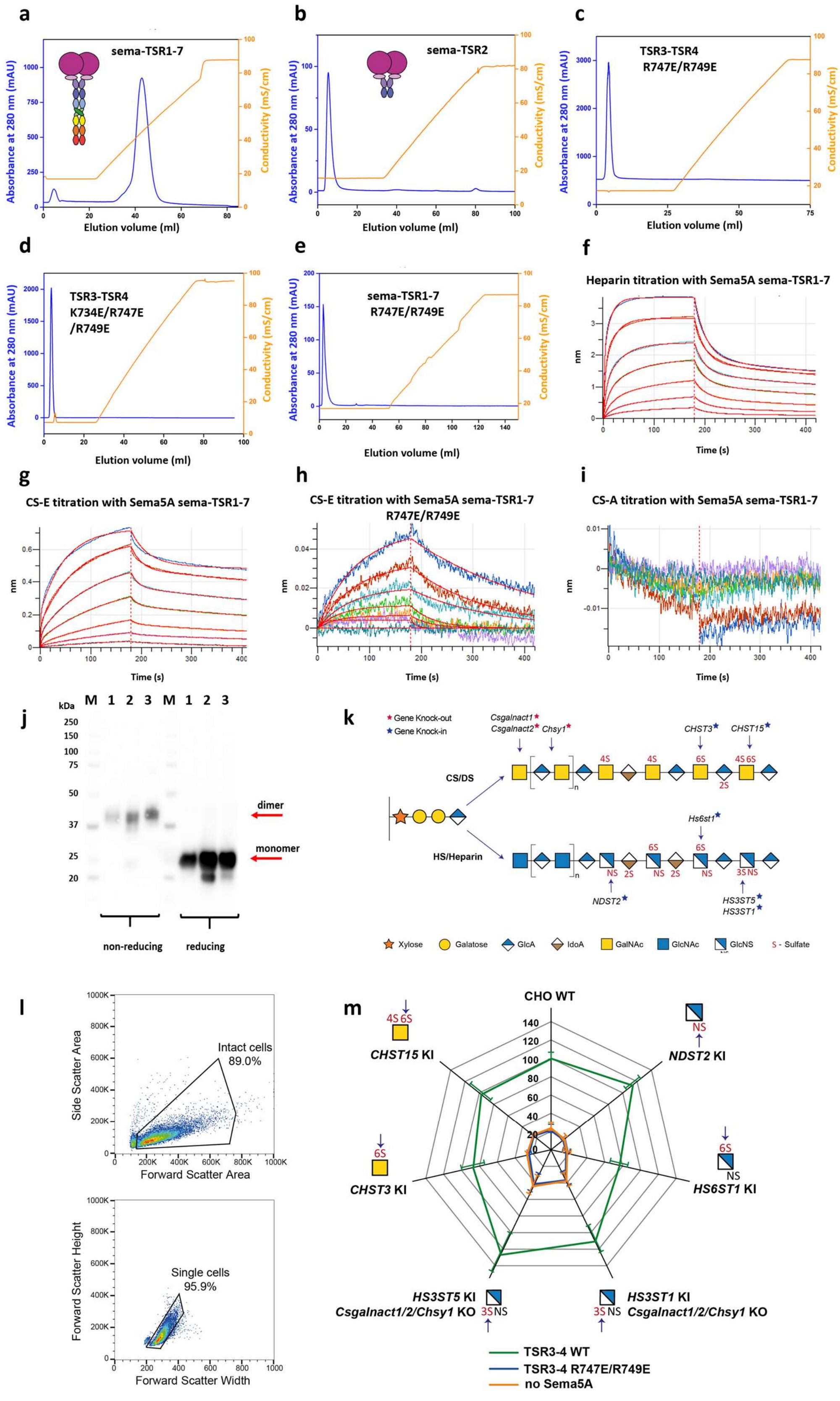

Supplementary Figure 1 Sema5A GAG specificity analysis

a-e Heparin affinity chromatography of Sema5A constructs. Purified proteins of **a,** $Sema5A_{sema-TSR1-7}$, **b,** $Sema5A_{sema-TSR2}$, **c,** $Sema5A_{TSR3-4}$ R747E/R749E, **d,** $Sema5A_{TSR3-4}$ K734E/R747E/R749E and **e,** $Sema5A_{sema-TSR1-7}$ R747E/R749E were subjected to a heparin affinity chromatography assay. Protein elution was followed by UV absorption at 280 nm (blue line) and the elution gradient is represented by the conductivity trace (orange line). $Sema5A_{sema-TSR1-7}$ was eluted with ~ 490 mM NaCl, 10 mM HEPES pH 7.5 (conductivity 45 mS/cm at the peak), whereas the other constructs did not bind to the column and was eluted with the loading buffer (~17 mS/cm). **f-h** Biolayer interferometry titration sensorgrams for characterization of Sema5A sema-TSR1-7 interaction with glycosaminoglycans; **f,** Sema5A WT with heparin, **g,** Sema5A WT with CS-E, **h,** Sema5A R747E,R749E with CS-E and **i,** Sema5A WT with CS-A. **j,** Western blot confirming biotinylation of Sema5A TSR3-4 and its mutants used for the GAGOme assay. Avi-tag biotinylated samples of 1: Sema5A TSR3-4-3C-Avi-His_6 wild type and its mutants 2: R747E/R749E and 3: K734E/R747E/R749E were loaded on SDS-PAGE under non-reducing and reducing conditions. Note the decrease in the apparent mass of sample bands under reducing conditions, consistent with the disulfide-linked dimer architecture of the construct. The blot was incubated with Streptactin HRP (BioRad) antibody at 1:25,000 dilution. **k,** A subset of the genes controlling HS and CS chain elongation and modifications that were engineered with KO (red star) or KI (blue star) in CHO cells and used in this study. **l,** Representative example (CHO WT cells) of the flow cytometry gating strategy used for dissection of $Sema5A_{TSR3-4}$ binding to GAGOme cell lines. Intact cells were gated as shown by plotting forward scatter area *versus* side scatter area. From the intact cell population, single cells were gated as shown by plotting forward scatter width *versus* forward scatter height. Mean fluorescence intensity (MFI) values for Alexa Fluor 488 was then determined for the intact singlet cells. All flow cytometry experiments were performed using the gating strategy shown here. **m,** $Sema5A_{TSR3-4}$ binding to genetically engineered CHO cell lines reveals GAG specificity of Sema5A. Source data are provided as a Source Data file.

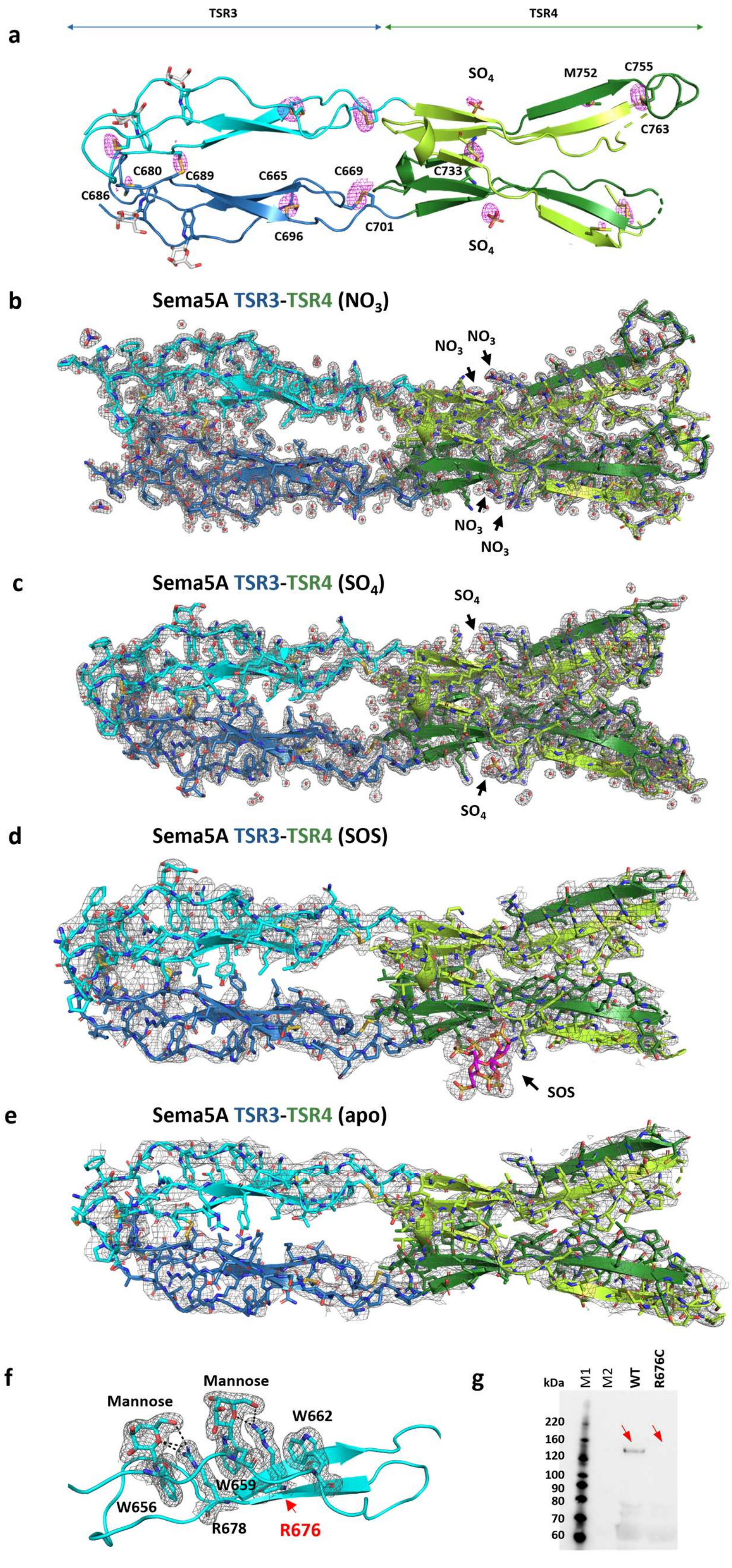

Supplementary Figure 2. Structure solution of Sema5A$_{TSR3-4}$.

a, Anomalous difference Fourier map (magenta mesh) calculated for the Sema5A$_{TSR3-4}$- SO_4 dimer in the crystallographic asymmetric unit contoured at 4σ and mapped on the final isomorphous refined model. The location of all 24 Cys or Met sulfur atoms, including disulfide bonds, were identified in the anomalous difference map, which proved to be instrumental for substructure determination and structure solution. Intriguing, the crystals used for S-SAD phasing grew from crystallization solutions containing sulfates, and an anomalous difference Fourier map peak at each binding site provided experimental confirmation of the sulfate ligand identity. **b-e,** Visualization of the sigma-weighted $2F_o$-F_c electron density map, shown at 1.0 RMSD contour level after the final round of refinement in phenix.refine for the Sema5A$_{TSR3-4}$-NO_3, Sema5A$_{TSR3-4}$-SO_4, Sema5A$_{TSR3-4}$-SOS, Sema5A$_{TSR3-4}$ apo structures, respectively. The Sema5A$_{TSR3-4}$-NO_3 structure contained several nitrate ions, not just at the active site, due to the high, 6 M nitrate concentration in the crystallization solution. Sema5A$_{TSR3-4}$-SO_4 contains a single sulfate ion at both active sites of the Sema5A$_{TSR3-4}$ dimer. Sema5A$_{TSR3-4}$-SOS contains a single SOS molecule per Sema5A$_{TSR3-4}$ dimer possibly due to limitations imposed by the crystal packing. **f,** Trp-ladder of TSR3 is stabilized by mannosyl-Trp/Arg H-bonds, visualized from the Sema5A$_{TSR3-4}$-NO_3 structure. We identified well resolved mannosyl residues in $^1C_4$ chair conformation with a C-glycosidic attachment of C1 of an α-mannose to the indole C2 of W656 and W659 residues. The hydroxyl groups of these mannosyl moieties engage in a hydrogen-bond network to stabilize the sidechain orientations within the Trp-Arg ladder, similarly to what was shown for BaI1[1] and Unc-5[2], which is expected to assist protein folding and secretion[3]. Residues are shown as sticks and the refined $2F_o$-F_c electron density map is shown at 1.0 RMSD contour level. H-bonds are indicated as dashed lines. R676 that is affected by a deleterious missense ASD related mutation p.R676C[4] is highlighted in red letters. **g,** Anti-His Western blot analysis of Sema5A$_{sema-\ TSR1-\ 7}$-His$_6$ (WT) and Sema5A$_{sema-\ TSR1-\ 7}$-His$_6$ R676C (R676C) small-scale protein expression, performed as described in [5]. Media of transiently transfected HEK293T cells 3 days post-transfection were loaded on SDS-PAGE under reducing conditions. M1: Benchmark Ladder (His-tagged proteins), M2: Benchmark Prestained Ladder (non-His-tagged). Source data are provided as a Source Data file.

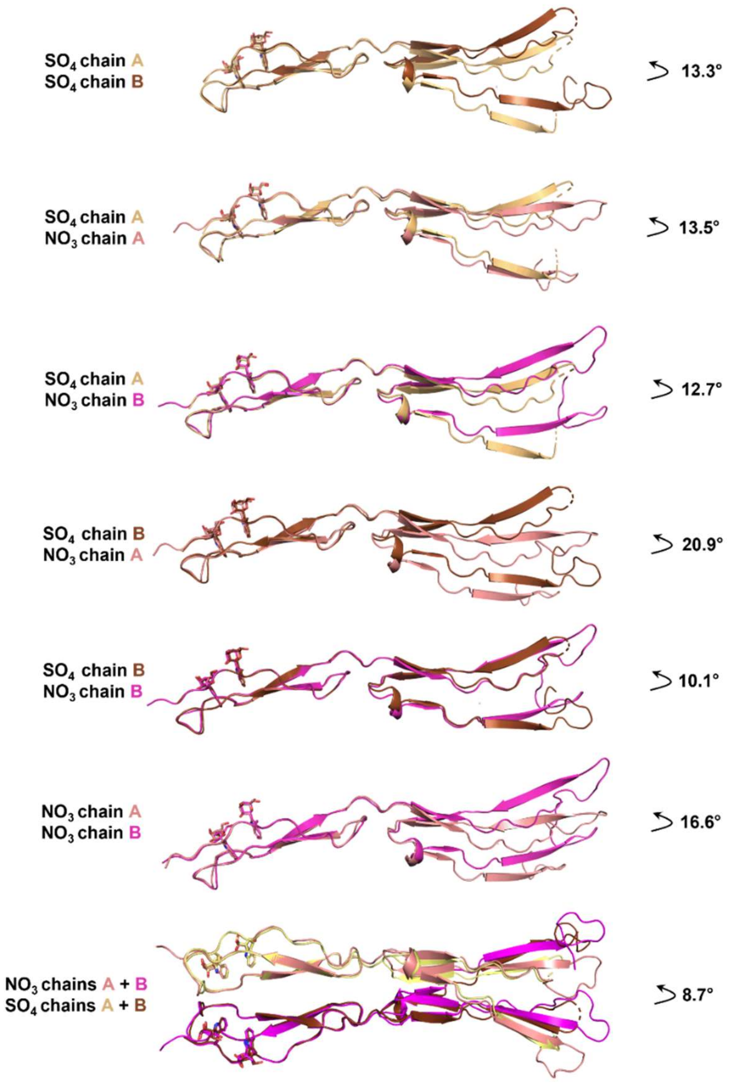

	TSR3				TSR4			
**Aligned protein chains**	**RMSD (Å)**	**# of Cα aligned**	**Translation (Å)**	**Rotation (°)**	**RMSD (Å)**	**# Cα aligned**	**Translation (Å)**	**Rotation (°)**
**SO_4-A; SO_4-B**	**0.60**	**49**	**-**	**-**	**0.38**	**46**	**0.41**	**13.3**
**SO_4-A; NO_3-A**	**0.49**	**49**	**-**	**-**	**0.77**	**46**	**0.47**	**13.5**
**SO_4-A; NO_3-B**	**0.55**	**49**	**-**	**-**	**0.85**	**46**	**0.08**	**12.7**
**NO_3-A; SO_4-B**	**0.68**	**49**	**-**	**-**	**0.91**	**50**	**0.89**	**20.9**
**SO_4-B; NO_3-B**	**0.36**	**49**	**-**	**-**	**0.98**	**50**	**0.81**	**10.1**
**NO_3-A; NO_3-B**	**0.67**	**51**	**-**	**-**	**0.57**	**52**	**0.70**	**16.6**
**NO_3-(A+B); SO_4-(A+B)**	**0.59**	**98**	**-**	**-**	**1.05**	**97**	**0.62**	**8.7**

Supplementary Figure. 3. Inter-domain hinge in Sema5A$_{TSR3-4}$

Monomer chains from Sema5A$_{TSR3-4}$-SO_4 and Sema5A$_{TSR3-4}$-NO_3 co-crystal structures, abbreviated here as SO_4-A/B or B or NO_3-A/B, respectively, were superposed based on the TSR3 domain by PDBeFOLD[6]. Domain translation, rotation and Cα atom root-mean-square deviation (RMSD) values were calculated in Pymol using the draw_rotation_axis.py script to compare Sema5A$_{TSR3-4}$ chains within and across crystal forms. The C-terminal epitope tag peptide (^{766}GTLEVLFQ^{773}) remaining after HRV 3C protease cleavage was neither included into the superimpositions nor visualized here.

Rank	PDB ID-chain	Protein name	Z score (similarity)	RMSD
1.	5FOE-B	GDP-fucose protein O-fucosyltransferase 2	6.4	1.8
2.	7R84-C	Brain-specific angiogenesis inhibitor 1 (BaI1)	5.6	2.4
3.	3R6B-A	Thrombospondin-1	5.4	2.0
4.	6RUS-B	Properdin	5.0	2.1
5.	1VEX-A	F-Spondin	4.9	2.1
6.	4E0S-B	Complement C5	4.6	1.9
7.	3OJY-B	Complement Component C8 Alpha Chain	4.4	2.8
8.	6RK1-A	CCN Family Member 3	4.4	3.1
9.	1SZL-A	F-Spondin	4.3	3.6
10.	4HQO-B	Sporozoite Surface Protein 2	4.2	2.2

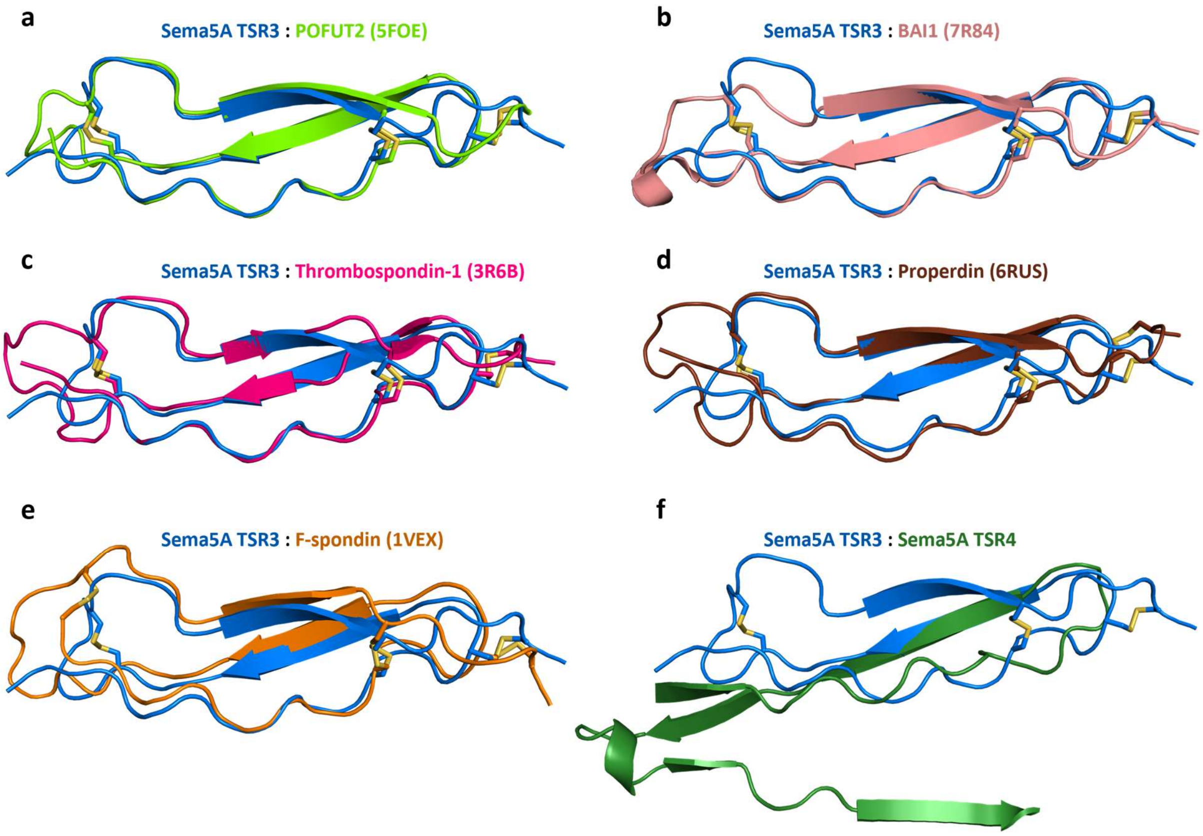

Supplementary Figure 4. Structural homologs of the Sema5A TSR3 domain.
Structural homology search for individual Sema5A TSR3 (Sema5A$_{TSR3-4}$-NO$_3$ chain residues 653-702) and TSR4 (Sema5A$_{TSR3-4}$-NO$_3$ chain A residues 703-754) domains was carried out using the DALI server[7] against the PDB50 database. Structure comparison data with the ten closest structural homologs of Sema5A TSR3 are shown. **a,-e,** Pairwise structural superimpositions of Sema5A TSR3 with the top five structural homolog domains from unrelated proteins. Disulfides stabilizing the TSR fold are shown as sticks. **f,** Structural superimposition of Sema5A TSR3 and TSR4 domains. Note that no structural homologs were retrieved for the TSR4 DALI50 query.

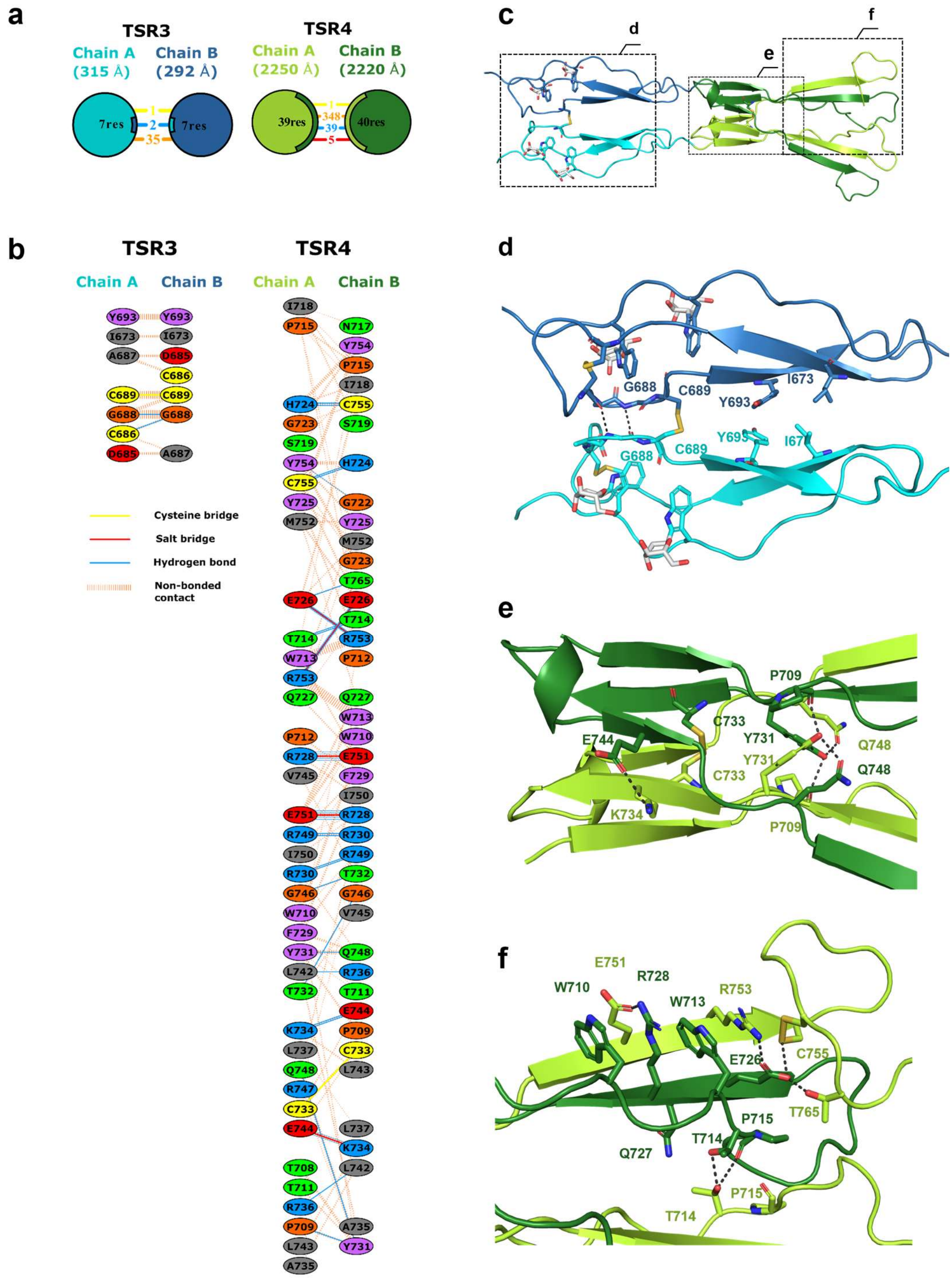

Supplementary Figure 5. Sema5A$_{TSR3-4}$ homodimer interactions.

a, and **b,** schematics of the Sema5A TSR3 and TSR4 homodimer interactions adapted from PDBSUM[8], based on the Sema5A$_{TSR3-4}$-NO_3 structure. The buried surface area of the respective protein chain is shown in brackets in **a**. Residue colours code in **b**: Positive (H,K,R); negative (D,E); S,T,N,Q = neutral; A,V,L,I,M = aliphatic; F,Y,W = aromatic; P,G = Pro&Gly; C = Cysteine. **c,** Cartoon presentation of Sema5A$_{TSR3-4}$ with close-up views in **d-f,** displaying key inter-chain interactions that together with the β-sheet hydrogen bond network stabilizes the dimer architecture. Colour coding as in **Fig. 1**.

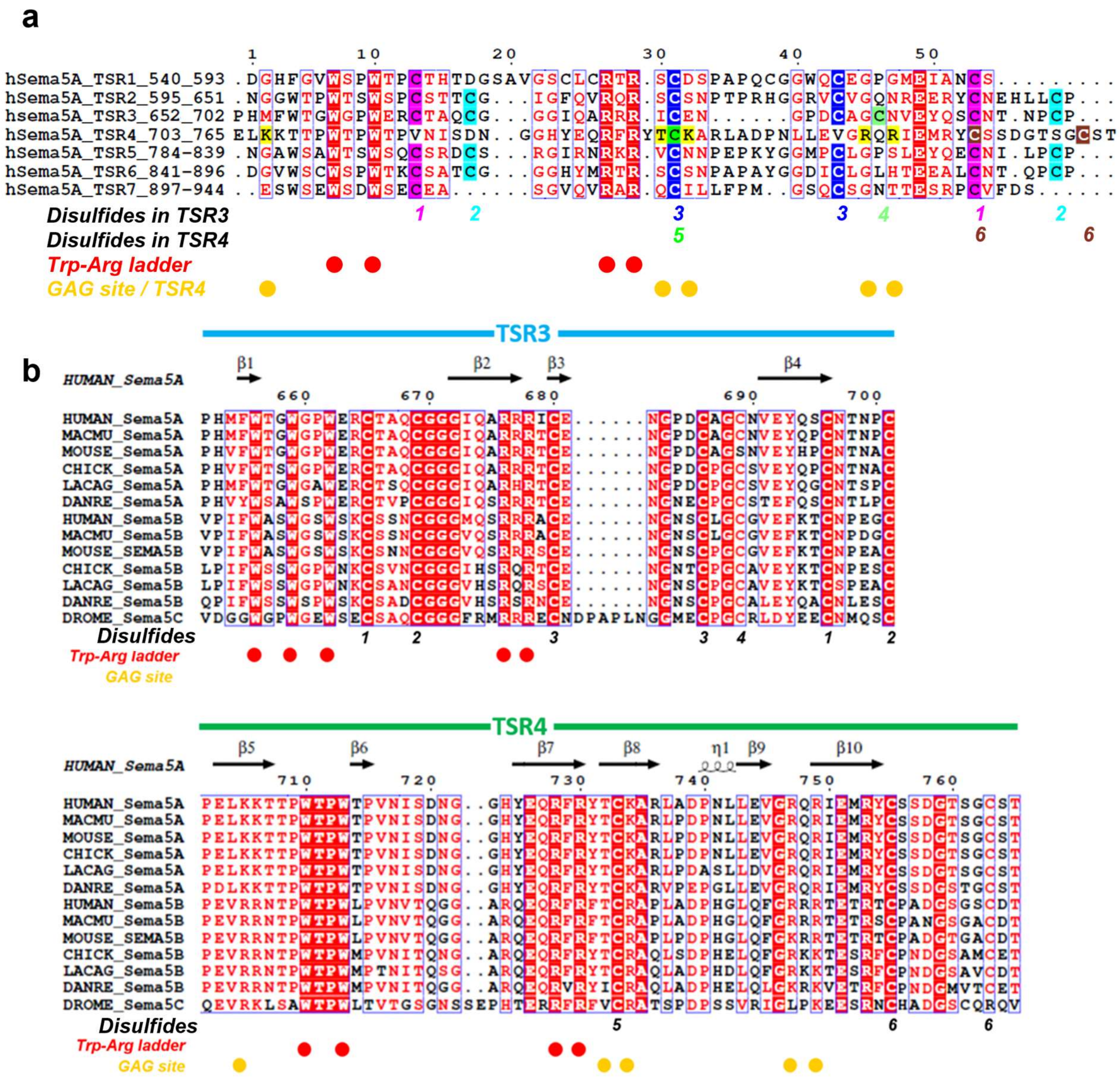

Supplementary Figure 6. Sequence conservation of Sema5A TSR domains

a, Sequence comparison of the human TSR1-7 domains. Sema5A residues that contribute to form either disulfide bonds, Trp-Arg ladder, or GAG site are indicated below the sequences. The Trp-Arg ladder is conserved in all 7 TSR repeats. TSR repeats no. 2,3,5,6,7 include W-x-x-W-x-x-W-x-x-C sequence motif that was identified as a canonical C-mannosylation motif for DPY19L1[9]. Among disulfide bridges that lock the β-strands of the TSR fold together (cf. Fig. 1f) #1 (pink) and #3 (blue) are conserved in all TSRs except TSR4 whereas TSRs 2,3,5,6 also feature disulfide bridge #2 (cyan). Note that no. 4 and 5 disulfide bonds covalently link TSR3 and 4 domains, respectively, from different monomers. The disulfide bridge conservation across TSR domains further indicates that the 3D domain swapped topology of TSR4, that is required for GAG site formation, is not shared with other TSRs within Sema5A. There is also no sequence conservation for the positively charged residues that constitute the GAG binding site of TSR4. **b,** TSR 3-4 domains are highly conserved within vertebrate class 5 semaphorins. Structure-based sequence alignment of the TSR 3-4 domain region from class 5 semaphorins.

Sequence numbering corresponds to human Sema5A. Domain regions and secondary structure elements of Sema5A$_{TSR3-4}$ are shown above the sequence with 3_{10} helix and beta strands represented as helix and arrows, respectively. Red shaded boxes enclose conserved positions and red letters show positions with homologous residues. Sema5A residues that form either disulfide bonds, Trp-Arg ladder and GAG site are indicated below the sequences. Note that no. 4 and 5 disulfide bonds covalently link TSR3 and 4 domains, respectively, from different monomers. Members of the semaphorin-5 class and their UniProt IDs (in order) in this alignment include: human Sema5A and Sema5B, Q13591 and Q9P283; *Macaca mulatta* (Rhesus macaque, MACMU) Sema5A and Sema5B, I2CT92 and NCBI RefSeq ID: XP_014985494.2; mouse Sema5A and Sema5B, Q62217 and Q60519; chicken Sema5A and Sema5B (CHICK), R4GFU3 and F1NSD7; *Lacerta agilis* (sand lizard, LACAG) Sema5A and Sema5B, NCBI RefSeq IDs XP_033009991.1 and XP_033018485.1; *Danio rerio* Sema5A and Sema5B (zebrafish, DANRE), F1R9L1 and F1QPQ6; fruit fly Sema5C, (DROME), Q9U631. Sequences were aligned using Clustal Omega and formatted using ESPript.

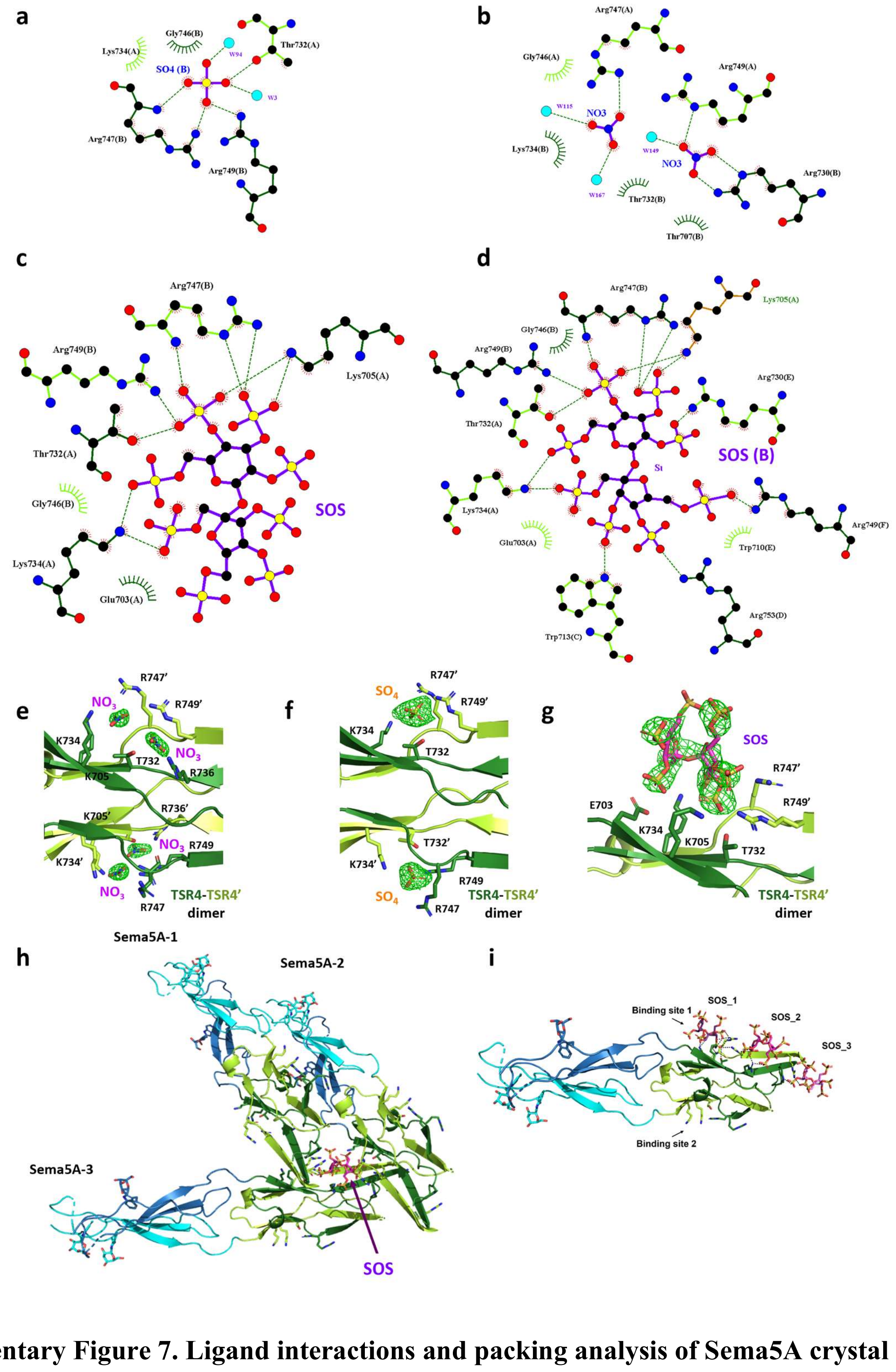

Supplementary Figure 7. Ligand interactions and packing analysis of Sema5A crystal structures.

Schematic representation of ligand interactions at the GAG site of Sema5A$_{TSR3-4}$ is shown for co-complexed structures with **a**, sulfate, **b,** nitrate and **c,** sucrose octasulfate (SOS) within the crystal asymmetry unit, adapted from LigPlot+[10]. Potential hydrogen bonds are shown as dashes. Note

that interactions for only nitrates that are coordinated at the GAG site are shown here. **d,** Schematic representation of interactions of a single SOS molecule with residues from three crystallographic copies of Sema5A$_{TSR3-4}$ dimers, adapted from LigPlot^{+}. Chain labels are shown in parentheses for each residue, with A&B, C&D and E&F each referring to a Sema5A$_{TSR3-4}$ dimer, respectively. SOS form additional interactions with Sema5A$_{TSR3-4}$ beyond the core GAG binding site that accommodates sulfate and nitrate ligands. Some of these contacts may be considered for providing auxiliary interactions to further improve binding affinity and specificity to a GAG interaction partner of Sema5A. **e-g,** Polder mF_{obs}-DF_{model} omit maps (green) contoured at 2.5 σ on crystallographic ligands in **e,** Sema5A$_{TSR3-4}$-SO_4 **f,** Sema5A$_{TSR3-4}$-NO_3 **g,** Sema5A$_{TSR3-4}$-SOS structures. **h,** A Sema5A$_{TSR3-4}$ dimer together with its two crystallographic copies coordinate together a single SOS molecule. **i,** A single Sema5A$_{TSR3-4}$ dimer forms crystal lattice contacts with three SOS molecules. Note that SOS molecules line up at binding site '1' while ligand binding at the opposite binding site '2' is precluded due to steric hindrance from crystal packing.

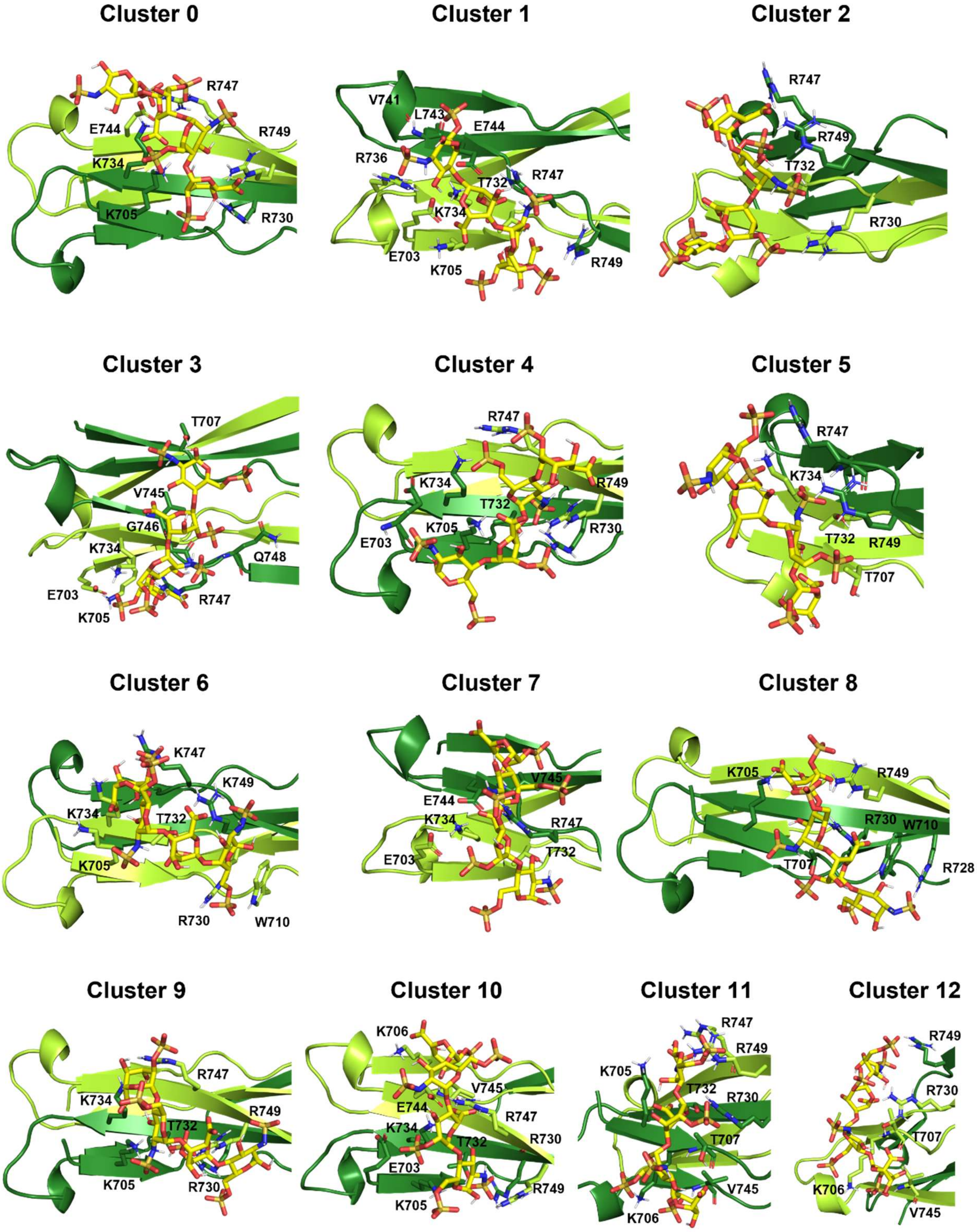

Supplementary Figure 8. Analysis of Sema5A *in silico* docking with heparin tetrasaccharides.

The *figure continues on next page.*

Cluster	Members	Representative	Weighted Score
0	527	Center	-778.4
		Lowest Energy	-846.3
1	408	Center	-792.4
		Lowest Energy	-843.6
2	341	Center	-388.2
		Lowest Energy	-804.7
3	150	Center	-779.2
		Lowest Energy	-829.3
4	103	Center	-770.6
		Lowest Energy	-804.2
5	94	Center	-379.0
		Lowest Energy	-779.9
6	41	Center	-382.4
		Lowest Energy	-771.8
7	41	Center	-782.7
		Lowest Energy	-801.5
8	39	Center	-777.6
		Lowest Energy	-794.3
9	33	Center	-384.9
		Lowest Energy	-397.0
10	18	Center	-786.2
		Lowest Energy	-801.3
11	4	Center	-766.1
		Lowest Energy	-766.1
12	1	Center	-381.9
		Lowest Energy	-381.9

Supplementary Figure 8. Analysis of Sema5A *in silico* docking with heparin tetrasaccharides

Close-up structural views of each docked IdoA(2S)-GlcNS(6S)-IdoA(2S)-GlcNS(6S) tetramer heparin oligosaccharide poses, highlighting protein residues that are forming hydrogen bonds with the ligand. ClusPro clusters retrieved 1,000 models using interface RMSD with a 9-Å radius and ranks the clusters based on cluster population. List of the clusters and their energy scores are shown.

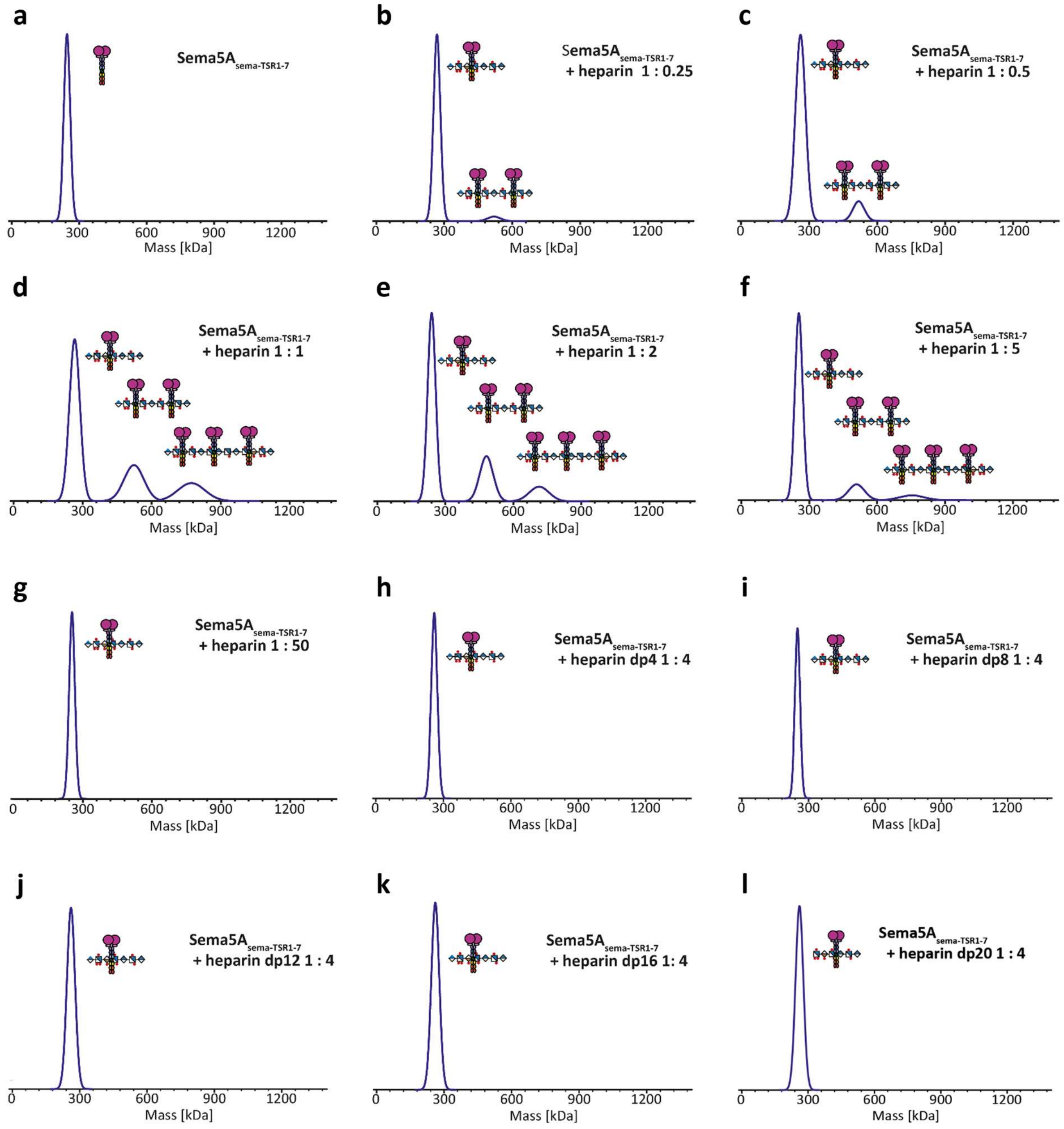

Supplementary Figure 9. Multimerization from Sema5A mixed with heparin or heparin oligosaccharides. Mass photometry mass distributions of **a-g,** 100 nM Sema5A$_{\text{sema-TSR1-7}}$ titrated with heparin at various concentrations, ratios of Sema5A was 1, and heparin was from 0.25 (25 nM) to 50-fold difference (5 µM). **h-l,** ratio 1 (100 nM) Sema5A$_{\text{sema-TSR1-7}}$: 4 (400 nM) size-defined heparin oligosaccharides. Blue lines represent the best-fit Gaussian distributions of the mass photometry data plotted with 8.3 kDa bin size of samples containing Sema5A$_{\text{sema-TSR1-7}}$ and heparin or heparin oligosaccharides. Source data are provided as a Source Data file.

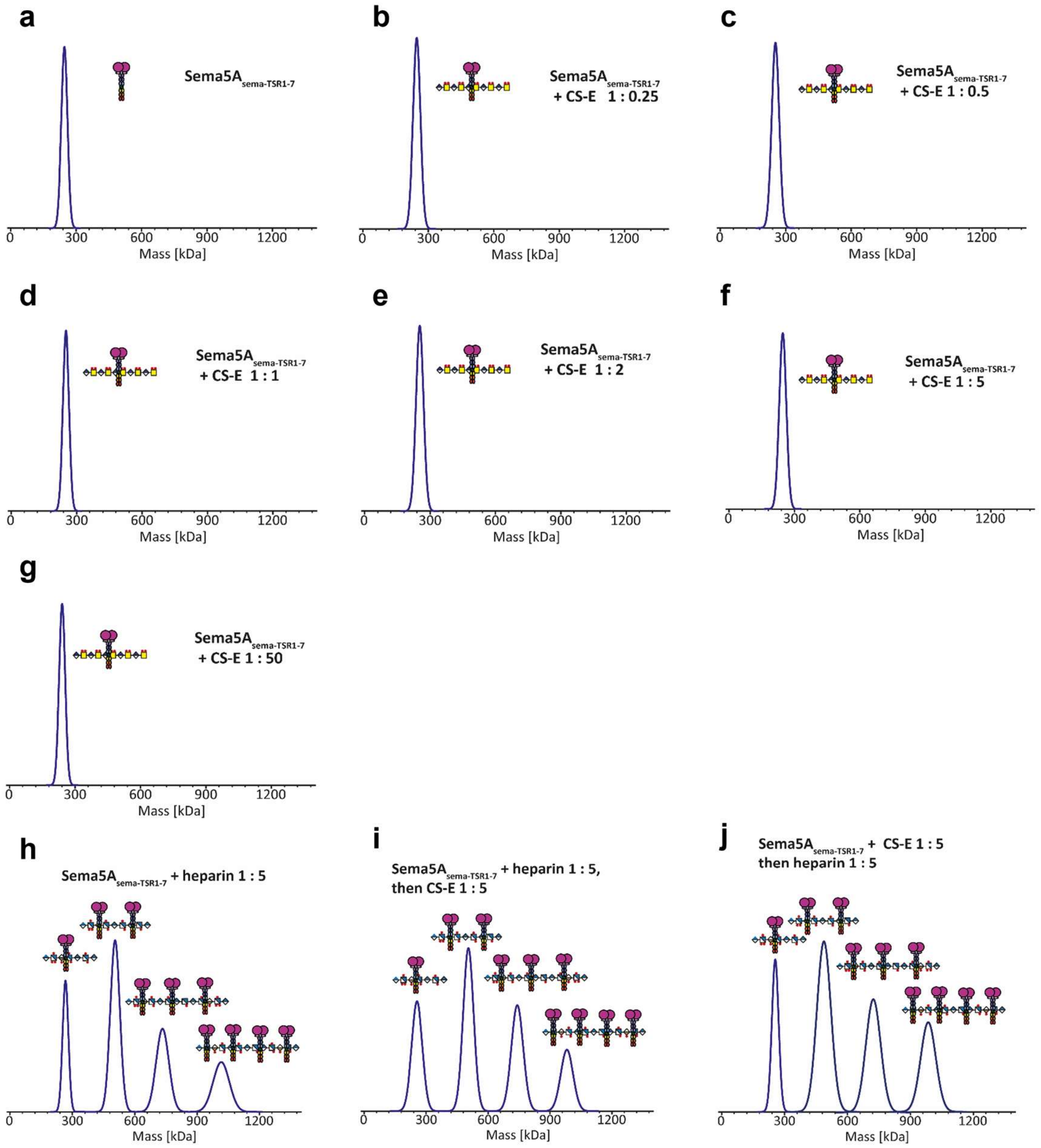

Supplementary Figure 10. CS-E does not multimerize Sema5A.

a-g Mass photometry mass distributions of 100 nM Sema5A$_{\text{sema-TSR1-7}}$ mixed with CS-E at various concentrations, ratios of Sema5A was 1, and CS-E was from 0.25 (25 nM) to 50-fold difference (5 µM). **h-j** CS-E does not suppress heparin-dependent Sema5A multimerization. **h,** Mass photometry mass distributions of 100 nM Sema5A$_{\text{sema-TSR1-7}}$ mixed with 500 nM heparin (a ratio of 1: 5 Sema5A to heparin). **i,** Mass photometry mass distributions of 100 nM Sema5A$_{\text{sema-TSR1-7}}$ pre-incubated with 500 nM heparin and then mixed with 500 nM CS-E (a ratio of 1: 5 Sema5A to heparin, and competed with equal GAG quantities of CS-E). **j,** Mass photometry mass distributions of 100 nM Sema5A$_{\text{sema-TSR1-7}}$ pre-incubated with 500 nM CS-E and then mixed with 500 nM heparin (a ratio of 1: 5 Sema5A to CS-E and competed with equal GAG quantities of heparin). Blue lines represent the best-fit Gaussian distributions of the mass photometry data plotted with 8.3 kDa bin size of samples containing Sema5A$_{\text{sema- TSR1- 7}}$, CS-E, and when applicable, heparin. Source data are provided as a Source Data file.

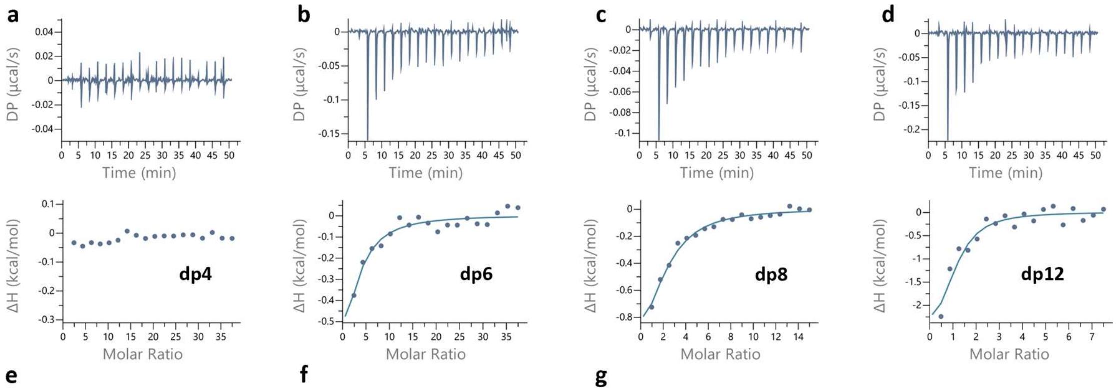

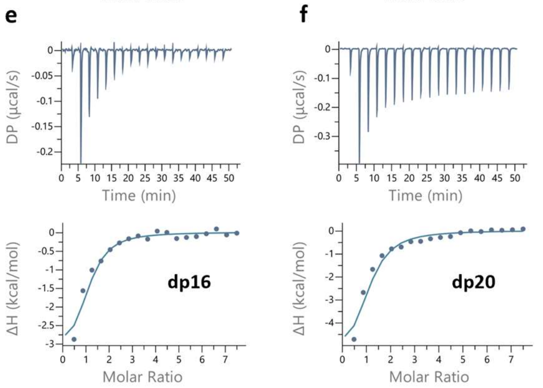

g

Cell	Syringe	N (fixed)	Kd (µM)	ΔH (kcal/mol)	-TΔS (kcal/mol)
Sema5A$_{sema-TSR1-7}$	dp4	n/a	n/a	n/a	n/a
Sema5A$_{sema-TSR1-7}$	dp6	2	41 ± 15	-1.53 ± 0.31	-4.45
Sema5A$_{sema-TSR1-7}$	dp8	2	15 ± 3	-1.44 ± 0.15	-5.13
Sema5A$_{sema-TSR1-7}$	dp12	1	3.8 ± 2.2	-3.10 ± 0.64	-4.3
Sema5A$_{sema-TSR1-7}$	dp16	1	2.3 ± 1.0	-3.46 ± 0.44	-4.24
Sema5A$_{sema-TSR1-7}$	dp20	1	2.8 ± 1.2	-5.99 ± 0.78	-1.59

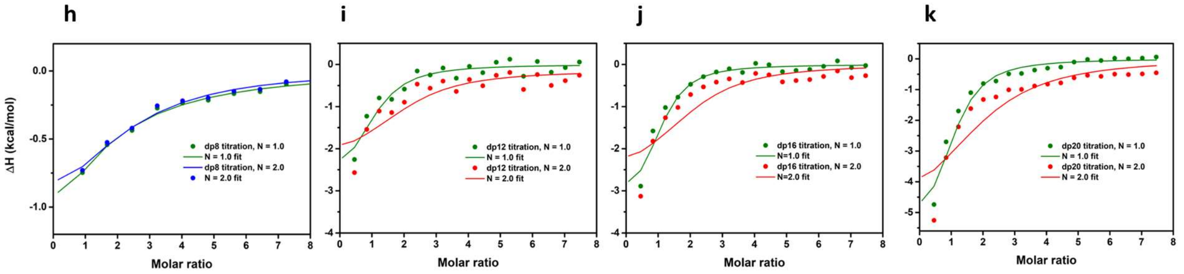

Supplementary Figure 11. ITC data for the binding of size-fractionated heparins to Sema5A$_{sema-TSR1-7}$. Representative ITC experiments (**a**-**f**) are shown with raw thermogram (thermal power versus time), at the top and binding isotherm (normalized heats versus molar ratio) at the bottom. **g,** Thermodynamic parameters from the interaction analyses. K_d: equilibrium dissociation constant (=1/K), ΔH: binding enthalpy, -TΔS: binding entropy. †: Not measurable under experimental conditions possibly due to low affinity interactions. Error values represent the error calculated through iteration fit of the data sets by the Microcal PEAQ-ITC analysis software. Note that during isotherm model fitting, we followed the low Wiseman c parameter ITC titration method and fixed the binding stoichiometry involving GAG sites per Sema5A dimer (N) as proposed in[11,12]. **h-k** Model comparisons for fitting ITC isotherms from Sema5A$_{sema-TSR1-7}$ titrations with size-defined heparins. Binding models assuming N=1.0 and N=2.0 fixed stoichiometries were fitted to Sema5A$_{sema-TSR1-7}$ calorimetry titrations with **a,** dp8; **b,** dp12; **c,** dp16; **d,** dp20, respectively. For dp8, these models gave very similar fits, thus the titration data is compatible with either one or two dp8 molecule binding to a Sema5A$_{sema-TSR1-7}$ dimer. For larger size-defined heparins, the titration data could be well fitted with an isotherm model assuming N=1 stoichiometry (green) but not with N=2 (red), indicating that a single Sema5A$_{sema-TSR1-7}$ dimer cannot bind more than one size-defined heparin molecule with a size larger than dp12. Source data are provided as a Source Data file.

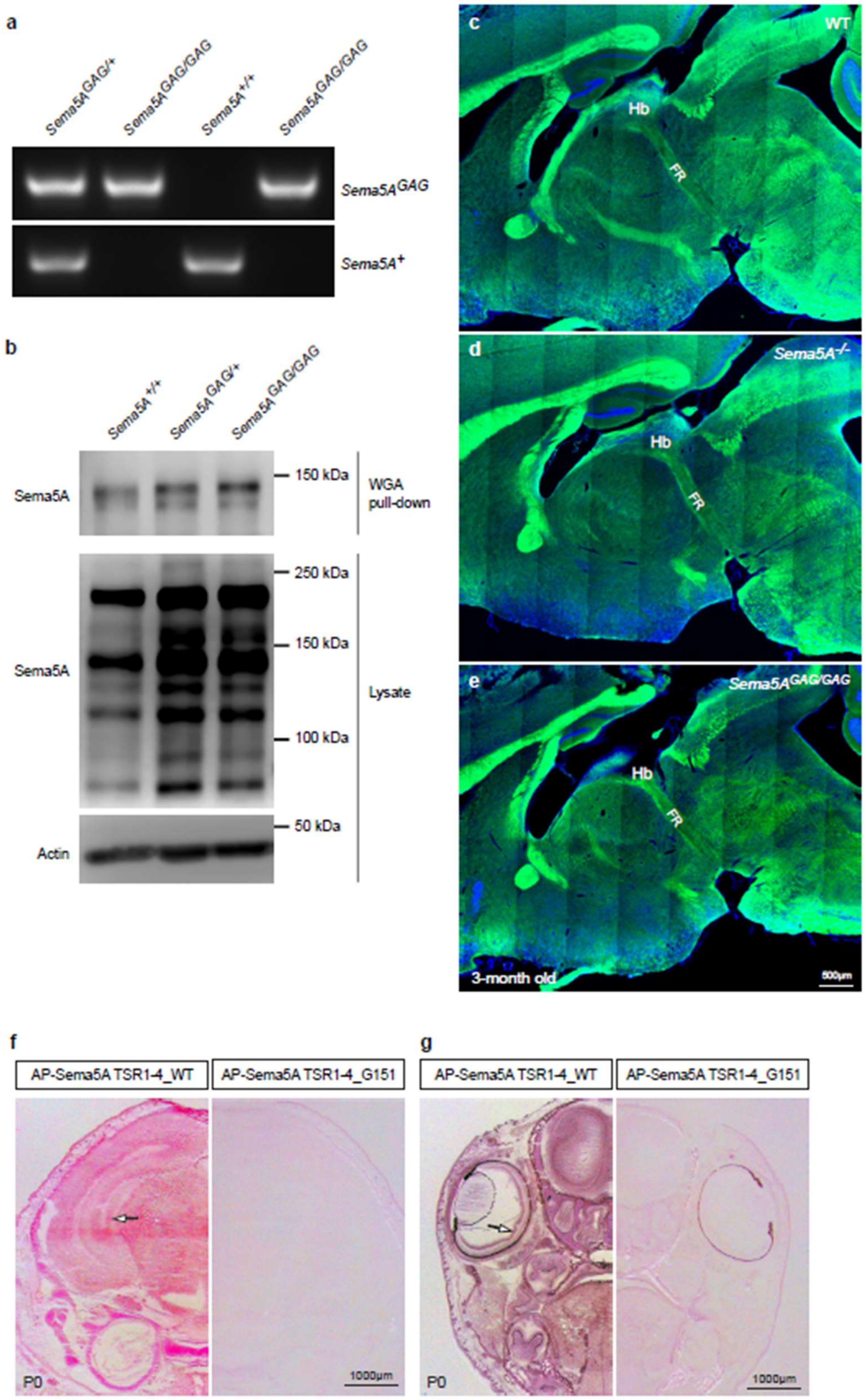

Supplementary Figure 12. Expression of Sema5A in *Sema5a*^{GAG} mice

a, Image of agarose gel showing Ethidium bromide stained PCR products amplified from *Sema5a*$^{GAG/+}$, *Sema5a*$^{GAG/GAG}$, *Sema5a*$^{+/+}$, and *Sema5a*$^{GAG/GAG}$ genomic DNA. The molecular weight of the PCR products is 293 bp. **b,** Western blot analysis of forebrain homogenates prepared from *Sema5a*$^{+/+}$, *Sema5a*$^{GAG/+}$, and *Sema5a*$^{GAG/GAG}$ P7 pups. WGA pull-down (WGA) of glycoproteins from brain lysates; Lysate: whole brain lysate before WGA pull-down. Blots were probed with anti-Sema5A or anti-actin to control for protein loading. **c-e** Sagittal sections of 3-month-old mouse brains stained with fluoromyelin (green) and DAPI (blue). Rostral is toward the left side. The habenula (Hb) in the dorsal thalamus and the fasciculus retroflexus (FR) are labeled. No obvious defects in FR fasciculation, thickness, or projection toward the interpeduncular nucleus in the midbrain were observed. Representative images of **c** wild-type, **d** *Sema5a-/-,* and **e** *Sema5a* $^{GAG/GAG}$ brains are shown (n= 3 per genotype). Scale bar, 500 µm. **f, g** Binding of recombinant alkaline phosphatase tagged Sema5A thrombospondin repeats 1-4 (AP-Sema5A-TSR1-4) harboring wildtype (WT) or GAG binding deficient (K734E,R747E,R749E, cf. Supplementary Fig. 1d) (G151) TSRs to coronal sections of the P0 mouse head. WT TSR1-4 binds strongly and broadly to tissue sections, including the developing hippocampus (arrow in **f**), and the inner retina (arrow in **g**). Scale bar, 1000 µm. Source data are provided as a Source Data file.

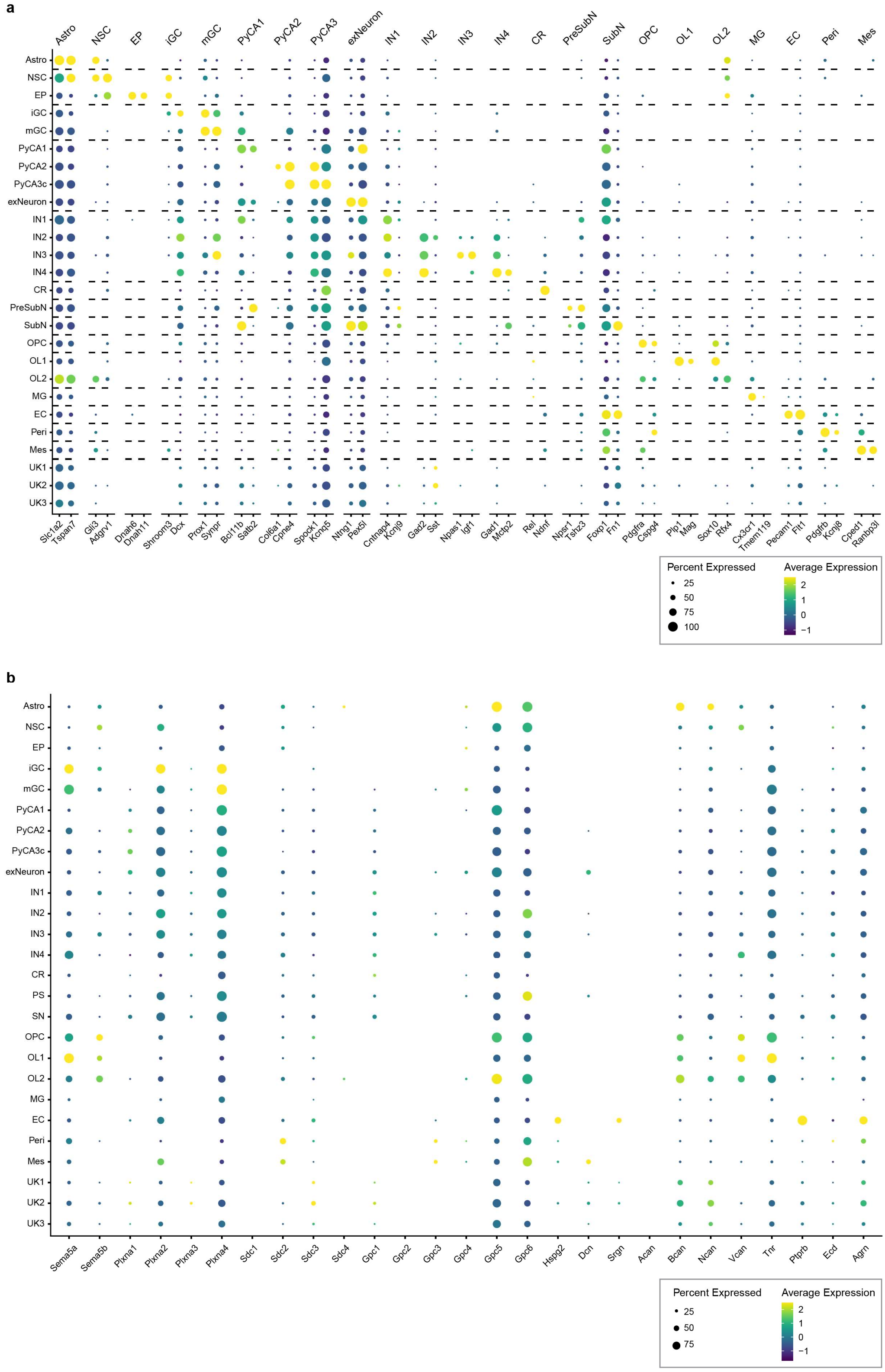

Supplementary Figure 13. Gene expression analysis at single nucleus resolution of the developing mouse hippocampus

a, Dot plots analysis of snRNAseq dataset of WT P10 with marker genes used for cell type identification. **b**, Dot plots analysis of genes of interest revels cell type specific distribution and expression levels. Gene expression levels are normalized to average gene expression (color coded calibration). For each cell cluster, the percentile of cells expressing a specific gene product is indicated by the dot size. The snRNAseq dataset (GEO: GSE186216, [13]) was re-analyzed. Astrocytes (Astro), neural stem cells (NSC), ependymal cells (EP), immature granulate cells (iGC), mature granule cells (mGC), pyramidal neuron (Py), Cornu Ammonis (CA), excitatory neurons, non-pyramidal (exNeuron), interneuron (IN), Cajal-Retzius cells (CR), presubicular neurons (PS), subicular neurons (SN), oligodendrocyte progenitor cell (OPC), microglia (MG), endothelial cells (EC), pericytes (Peri), mesenchymal cells (Mes), unknown cluster (UK).

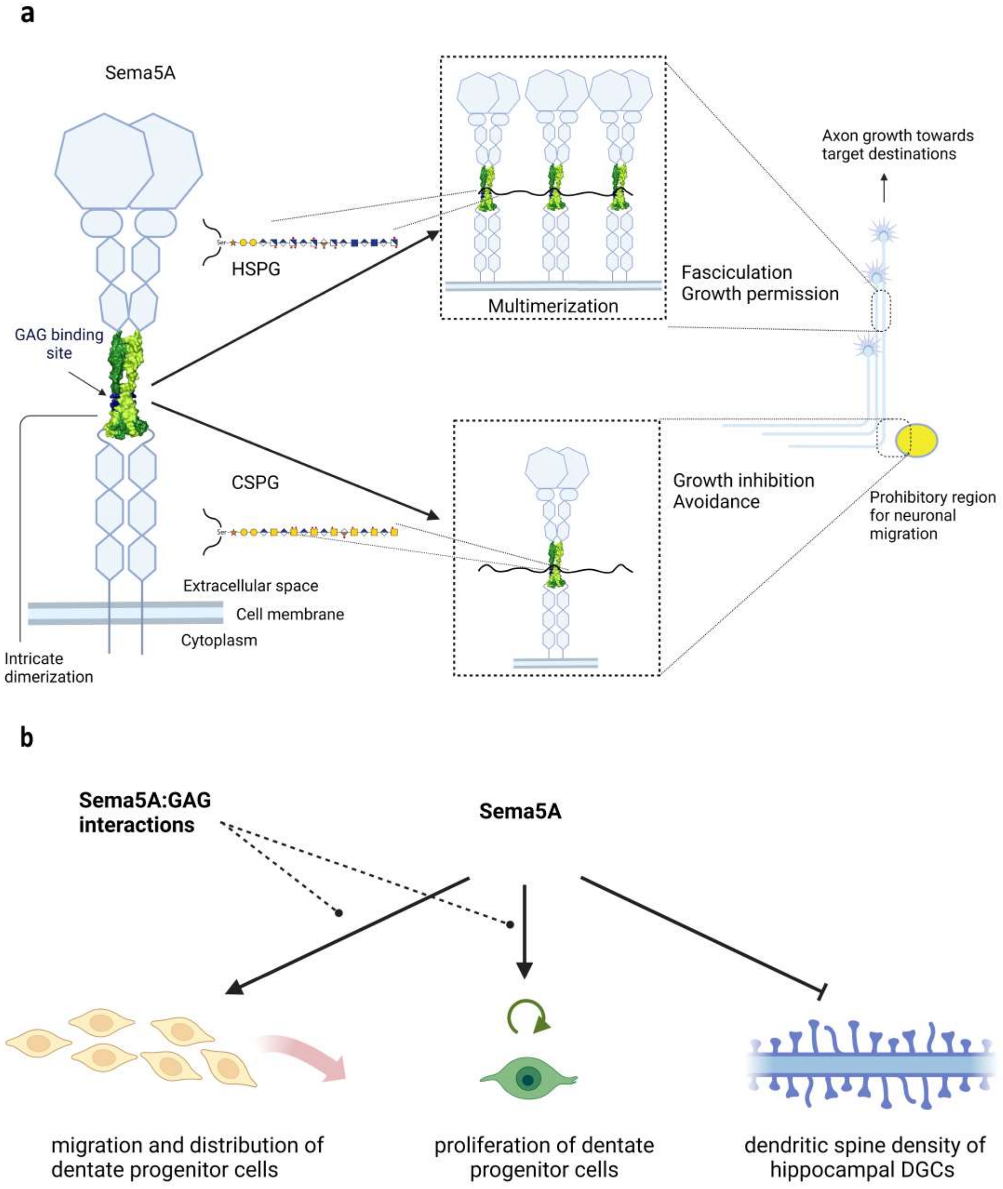

Supplementary Figure 14. Schematic depiction of Sema5A-GAG interactions and function in neuronal development.
Our study resolves the structural basis for Sema5A dimerization, uncovers HS-GAG mediated multimerization, and reveals context-dependent functions of this interaction in the developing hippocampus. **a,** Structural data identify a domain-swapped TSR4 dimer that covalently locks Sema5A into a dimer. Furthermore, TSR4 harbors the core of a GAG-binding module that supports the binding of both HS and CS-GAGs. Glycomics studies revealed that Sema5A preferentially interacts with highly sulfated HS epitopes featuring NS, 2S, and 6S modifications. HS-GAG binding to TSR4 induces Sema5A multimerization, while CS-GAG fails to induce multimerization. Specific Sema5A-GAG molecular interactions and HS-GAG dependent Sema5A multimerization may explain how Sema5A either inhibit neuronal migration or promote axon growth and fasciculation depending on the GAG context. Illustration is created with BioRender.com. **b,** Functional studies with GAG-binding-deficient Sema5A revealed

significance in progenitor cell migration but not in the regulation of dendritic spine density. We provide evidence that GAG-binding-deficient Sema5A functions as a null allele in migrating granule cells. We propose that multimerization of Sema5A on the surface of migrating progenitor cells influences adhesive and repulsive interactions, molecular events important for directed cell migration. Illustration is created with BioRender.com.

Supplementary Tables

Supplementary Table 1. X-ray diffraction data collection and refinement statistics.

Dataset	**Sema5A$_{TSR3\text{-}4}$-SO_4 S-SAD**	**Sema5A$_{TSR3\text{-}4}$-SO_4 (PDB: 8CKG)**	**Sema5A$_{TSR3\text{-}4}$-NO_3 (PDB: 8CKK)**	**Sema5A$_{TSR3\text{-}4}$ apo (PDB: 8CKM)**	**Sema5A$_{TSR3\text{-}4}$-SOS (PDB: 8CKL)**
**Data collection**	anomalous dataset	native dataset	native dataset	native dataset	native dataset
Beamline	DLS-I23	DLS-I24	DLS-I24	DLS-I24	DLS-I03
Ligand	SO_4	SO_4	NO_3	apo	SOS
Software	XDS/ Aimless	AutoPROC/ STARANIZO	AutoPROC/ STARANIZO	XDS	AutoPROC/ STARANIZO
Reflection scaling method		Anisotropy corrected	Anisotropy corrected		Anisotropy corrected
Wavelength (Å)	2.7552	0.9686	0.9686	0.9999	0.97625
Resolution range (Å)	88.70 - 2.3 (2.38 - 2.3)	88.93 - 1.714 (1.855 - 1.714)	42.75 - 1.557 (1.768 - 1.557)	51.4 - 2.72 (2.77 - 2.72)	41.45 - 2.561 (2.841 – 2.561)
Diffraction limit (Å) along principal axes (in parantheses) of fitted ellipsoid		1.75 (0.999a*+0.054c*)<br>1.64, b*<br>2.21 (-0.27a*+0.963c*)	1.94, a*<br>1.53, b*<br>2.18, c*		2.625 (0.993a*-0.118c*),<br>2.42, b*,<br>3.919 (-0.029a*+c*)
Space group	C 1 2 1	C 1 2 1	P 21 21 21	C 1 2 1	C 1 2 1
Cell dimensions a, b, c (Å) α, β, γ (°)	105.31, 29.25, 90.7<br>90, 100.01, 90	105.1, 29.3, 90.4<br>90, 100.5, 90	51.9, 68.3, 85.5<br>90, 90, 90	104.1, 27.7, 92.3<br>90, 99.1, 90	106.1, 30.8, 84.2<br>90, 100.0, 90
Total reflections	226527 (17027)	130867 (3864)	310381 (14907)	44611 (3612)	32914 (1661)
Unique reflections	10568 (854)	20985 (1050)	24530 (1226)	7316 (382)	5008 (250)
Multiplicity	21.4 (19.9)	6.2 (3.7)	12.7 (12.2)	6.1 (5.0)	6.6 (6.6)
Completeness, spherical (%)	85.6 (71.8)	70.7 (16.9)	55.4 (8.9)	98.09 (86.87)	55.9 (10.7)
Completeness, ellipsoidal (%)		89.8 (46.1)	93.8 (72.8)		84.3 (36.6)
Mean I/sigma(I)	17.2 (0.8)	9.3 (1.0)	11.2 (1.9)	7.1 (0.3)	8.2 (0.8)
Wilson B-factor	56.16	29.3	22.67	99.19	73.54
R-merge	0.114 (3.673)	0.096 (1.200)	0.123 (1.564)	0.1335 (5.029)	0.143 (2.670)
R-meas	0.117 (3.769)	0.105 (1.400)	0.128 (1.633)	0.1465 (5.63)	0.156 (2.889)
R-pim	0.024 (0.807)	0.041 (0.700)	0.036 (0.465)	0.05928 (2.46)	0.061 (1.091)
CC1/2	0.999 (0.561)	0.995 (0.338)	0.996 (0.624)	0.994 (0.41)	0.994 (0.098)
**Refinement**					
No. unique reflections in refinement	10478 (821)	20984 (146)	24526 (141)	7192 (635)	4996
R-work / R-free	0.3814 / 0.4374	0.2060 / 0.2312	0.1966 / 0.2292	0.2878 / 0.3311	0.2554 / 0.2850
No. non-hydrogen atoms		1806	2098	1571	1614
Protein residues		209	234	205	197
RMSDs					
bond length (Å)		0.007	0.003	0.003	0.002
bond angles (°)		0.93	0.70	0.55	0.64
Ramachandran favored (%)		96.0	96.09	95.43	98.40
Ramachandran allowed (%)		4.0	3.91	4.57	1.60
Ramachandran outliers (%)		0	0	0	0
Rotamer outliers (%)		0	0	0	0
Clashscore		2.77	2.48	3.02	6.25
Average B-factor		51.31	35.52	146.20	82.3

Values in parentheses correspond to the highest resolution shell unless otherwise stated.
RMSD: Root Mean Square Deviations.
Asterisks (*) denote reciprocal space.

Supplementary Table 2 ssDNA oligonucleotides used in this study.

Primer name	Primer sequence 5'-3'	Background
Sema5A_E23_Fwd	CATGTCCGGAGAGGCCCAGGGTACG	Forward primer to clone Sema5A sema-TSR1-7
Sema5A_S944_Rev	CAGTCGTACGAGAGTCAAACACACACGGCCG	Reverse primer to clone Sema5A sema-TSR1-7
Sema5A_EcoRI_Fwd	CCGTCTCAGGCCGAATTCAAGCT	Forward primer to clone Sema5A sema-TSR2
Sema5A_P651_Rev	GATCCGTACGGGGACATAGCAAATGTTCATTGCAGTATCTTTCCTC	Reverse primer to clone Sema5A sema-TSR2
Sema5A_P652_Fwd	GATCACCGGTCCACACATGTTCTGGACAGGCTGG	Forward primer to clone Sema5A TSR3-TSR4
Sema5A_T765_Rev	GATCGGTACCTGTGGAGCAGCCACTGGTGC	Reverse primer to clone Sema5A TSR3-TSR4
Sema5A_K734_For	CACTATGAGCAACGATTCCGATACACATGCGAAGCCCGCCTGGCTGATC	Forward primer to introduce K734E mutation to Sema5A *via* overlap extension PCR
Sema5A_K734_Rev	GATCAGCCAGGCGGGCTTCGCATGTGTATCGGAATCGTTGCTCATAGTG	Reverse primer to introduce K734E mutation to Sema5A *via* overlap extension PCR
Sema5A R747E_R749E_Fwd	CTGATCCGAATTTGCTGGAAGTGGGAGAACAGGAAATCGAAATGCGGTACTGTTCTAGCGAC	Forward primer to introduce R747E/R749E mutations to Sema5A *via* overlap extension PCR
Sema5A_R747E_R749E_Rev	GTCGCTAGAACAGTACCGCATTTCGATTTCCTGTTCTCCCACTTCCAGCAAATTCGGATCAG	Reverse primer to introduce R747E/R749E mutations to Sema5A *via* overlap extension PCR
ratSema5A_K734E_R747E_R749E_Fwd	gagGCTCGCCTGCCAGATCCAAACTTATTGGAAGTAGGAgagCAAgagATAGAAATGCGGTACTGTTCTAGTGATGGAACCAG	Forward primer to introduce K734E/R747E/R749E mutations to AP-ratSema5A TSR1-4 *via* overlap extension PCR
ratSema5A_K734E_ R747E_R749E_Rev	ctcTTGctcTCCTACTTCCAATAAGTTTGGATCTGGCAGGCGAGCctcACAGGTGTAGCGGAACCGCTG	Reverse primer to introduce K734E/R747E/R749E mutations to AP-ratSema5A TSR1-4 *via* overlap extension PCR
ratSema5A Xma1_Fwd	CAGTCCCGGGTTCCGGAAGATCTCCG	Forward primer to clone AP-ratSema5A TSR1-4 K734E_R747E_R749E into pcDNA 1.1
ratSema5A Hpa1_Rev	GATCGTTAACAACAACAATTGCATTCATTTTATGTTTCAGGTTCAGG	Reverse primer to clone AP-ratSema5A TSR1-4 K734E_R747E_R749E into pcDNA 1.1

Supplementary References

1 Wang, J. *et al.* RTN4/NoGo-receptor binding to BAI adhesion-GPCRs regulates neuronal development. *Cell* **184**, 5869-5885 e5825, doi:10.1016/j.cell.2021.10.016 (2021).

2 Akkermans, O. *et al.* GPC3-Unc5 receptor complex structure and role in cell migration. *Cell* **185**, 3931-3949 e3926, doi:10.1016/j.cell.2022.09.025 (2022).

3 Shcherbakova, A. *et al.* C-mannosylation supports folding and enhances stability of thrombospondin repeats. *Elife* **8**, doi:10.7554/eLife.52978 (2019).

4 Mosca-Boidron, A. L. *et al.* A de novo microdeletion of SEMA5A in a boy with autism spectrum disorder and intellectual disability. *Eur J Hum Genet* **24**, 838-843, doi:10.1038/ejhg.2015.211 (2016).

5 Aricescu, A. R., Lu, W. & Jones, E. Y. A time- and cost-efficient system for high-level protein production in mammalian cells. *Acta Crystallogr D Biol Crystallogr* **62**, 1243-1250, doi:10.1107/S0907444906029799 (2006).

6 Krissinel, E. & Henrick, K. Secondary-structure matching (SSM), a new tool for fast protein structure alignment in three dimensions. *Acta Crystallogr D Biol Crystallogr* **60**, 2256-2268, doi:10.1107/S0907444904026460 (2004).

7 Holm, L. Dali server: structural unification of protein families. *Nucleic Acids Res*, doi:10.1093/nar/gkac387 (2022).

8 Laskowski, R. A., Jablonska, J., Pravda, L., Varekova, R. S. & Thornton, J. M. PDBsum: Structural summaries of PDB entries. *Protein Sci* **27**, 129-134, doi:10.1002/pro.3289 (2018).

9 Minakata, S. *et al.* Protein C-Mannosylation and C-Mannosyl Tryptophan in Chemical Biology and Medicine. *Molecules* **26**, doi:10.3390/molecules26175258 (2021).

10 Laskowski, R. A. & Swindells, M. B. LigPlot+: multiple ligand-protein interaction diagrams for drug discovery. *J Chem Inf Model* **51**, 2778-2786, doi:10.1021/ci200227u (2011).

11 Tellinghuisen, J. Isothermal titration calorimetry at very low c. *Anal Biochem* **373**, 395-397, doi:10.1016/j.ab.2007.08.039 (2008).

12 Turnbull, W. B. & Daranas, A. H. On the value of c: can low affinity systems be studied by isothermal titration calorimetry? *J Am Chem Soc* **125**, 14859-14866, doi:10.1021/ja036166s (2003).

13 Chen, K. *et al.* Enhanced hippocampal neurogenesis mediated by PGC-1alpha-activated OXPHOS after neonatal low-dose Propofol exposure. *Front Aging Neurosci* **14**, 925728, doi:10.3389/fnagi.2022.925728 (2022).
