# Supplementary material for: Structure and function of Semaphorin-5A glycosaminoglycan interactions
Source: Nat Commun. 2024 Mar 28;15:2723. doi: 10.1038/s41467-024-46725-7 (PMC10978931; doi:10.1038/s41467-024-46725-7)
Supplement: Supplementary file 2 — Reporting Summary [file 41467_2024_46725_MOESM2_ESM.pdf]

nature portfolio

Corresponding author(s): E Yvonne Jones
Roman J Giger
Rebecca L Miller
Gergely N Nagy

Last updated by author(s): 15/02/24

Reporting Summary

Nature Portfolio wishes to improve the reproducibility of the work that we publish. This form provides structure for consistency and transparency in reporting. For further information on Nature Portfolio policies, see our Editorial Policies and the Editorial Policy Checklist.

Statistics

For all statistical analyses, confirm that the following items are present in the figure legend, table legend, main text, or Methods section.

n/a	Confirmed	
☐	☒	The exact sample size (*n*) for each experimental group/condition, given as a discrete number and unit of measurement
☐	☒	A statement on whether measurements were taken from distinct samples or whether the same sample was measured repeatedly
☐	☒	The statistical test(s) used AND whether they are one- or two-sided<br>*Only common tests should be described solely by name; describe more complex techniques in the Methods section.*
☒	☐	A description of all covariates tested
☒	☐	A description of any assumptions or corrections, such as tests of normality and adjustment for multiple comparisons
☐	☒	A full description of the statistical parameters including central tendency (e.g. means) or other basic estimates (e.g. regression coefficient) AND variation (e.g. standard deviation) or associated estimates of uncertainty (e.g. confidence intervals)
☐	☒	For null hypothesis testing, the test statistic (e.g. *F*, *t*, *r*) with confidence intervals, effect sizes, degrees of freedom and *P* value noted<br>*Give P values as exact values whenever suitable.*
☒	☐	For Bayesian analysis, information on the choice of priors and Markov chain Monte Carlo settings
☒	☐	For hierarchical and complex designs, identification of the appropriate level for tests and full reporting of outcomes
☒	☐	Estimates of effect sizes (e.g. Cohen's *d*, Pearson's *r*), indicating how they were calculated

Our web collection on statistics for biologists contains articles on many of the points above.

Software and code

Policy information about availability of computer code

Data collection	Anomalous diffraction data obtained for the Sema5ATSR3-4-SO4 crystals were integrated with XDS (version 20200131) and merged with XSCALE. Integrated data were obtained for the Sema5ATSR3-4-NO3, Sema5ATSR3-4-SO4 (high-resolution), Sema5ATSR3-4-SOS datasets from the AutoPROC (version 1.0.5) Diamond beamline data auto processing pipeline, implementing XDS (version 20200417) for data reduction and AIMLESS (version 0.7.4) for scaling. Diffraction data obtained for the Sema5ATSR3-4-apo crystals were integrated with XDS (version 20200417) within the diamond beamline auto processing pipeline and merged with XSCALE.
Data analysis	For the anomalous Sema5ATSR3-4-SO4 dataset, the CCP4 7.0.078 suite was used for experimental structure solution by running Crank2 pipeline for experimental phasing of sulfur single-wavelength anomalous diffraction (S- SAD) data, PRASA for substructure detection and Buccaneer for subsequent automated model building. Anisotropic cut-off was performed by STARANIZO (version 2.3.52) implemented in the Diamond beamline data autoBBprocessing pipeline for the Sema5ATSR3-4-NO3, Sema5ATSR3-4-SO4 (high-resolution), Sema5ATSR3-4-SOS datasets. Molecular replacement and structure refinement of all native datasets was performed using the PHENIX software package (version 1.20.1-4487). Manual model building was done in Coot (version 0.9.6).<br>Stereochemical properties were assessed in MOLPROBITY (version 4.02b-467). Superpositions were calculated using PDBeFOLD (version 2.58) and Pymol (version 2.4, Schrödinger, LLC), electrostatic potentials were generated using APBS web server (version 3.0). Buried surface areas of protein–protein interactions were calculated using the PDBsum web server (version 2022). Sequence alignments were generated with Clustal Omega (version 1.2.4), and were further edited by Jalview23. Structural homologs were identified with the DALI webserver (version 2022) Figures were produced with PyMOL (version 2.4, Schrödinger LLC), ESPRIPT (version 3.0) and BioRender (version 2023).<br>The ClusPro web server (version 2.0) was used for rigid body docking in the fully automated heparin docking mode.<br>The ColabFold notebook (version 1.5.2) was used for structural modelling.<br>BioLayer Interferometry data were were processed and analysed using the Octet Data Analysis HT software (version 11.1). The equilibrium

binding data were fitted with a Langmuir 1:1 binding isotherm and visualized in OriginPro 9.1.
Mass photometry data were collected and analyzed using the AcquireMP software (version 2023 R1.1, Refeyn)
Collected chromatograms and MALS data were analyzed and processed using the ASTRA6 software (version 6.1, Wyatt).
Isothermal titration calorimetry data were analyzed using a PEAQ-ITC analysis software (version 1.21) and further visualized using OriginPro 9.1.
ZEN software (version ZEN 2, Blue Edition) was used for image tiling and stitching.
BrdU+ cells in the mice dentate gyrus were quantified using ImageJ (version 1.51).
GraphPad Prism (version 8) was used for statistical analysis of mouse genetic data.

For manuscripts utilizing custom algorithms or software that are central to the research but not yet described in published literature, software must be made available to editors and reviewers. We strongly encourage code deposition in a community repository (e.g. GitHub). See the Nature Portfolio guidelines for submitting code & software for further information.

Data

Policy information about availability of data

All manuscripts must include a data availability statement. This statement should provide the following information, where applicable:

- Accession codes, unique identifiers, or web links for publicly available datasets
- A description of any restrictions on data availability
- For clinical datasets or third party data, please ensure that the statement adheres to our policy

Coordinates and structure factors have been deposited in the Protein Data Bank with accession numbers: 8CKG (Sema5ATSR3-4 complexed with sulfate; https://doi.org/10.2210/pdb8ckg/pdb), 8CKK (Sema5ATSR3-4 complexed with nitrate, https://doi.org/10.2210/pdb8ckk/pdb), 8CKL (Sema5ATSR3-4 complexed with sucrose octasulfate, https://doi.org/10.2210/pdb8ckl/pdb), and 8CKM (Sema5ATSR3-4, unliganded state, https://doi.org/10.2210/pdb8ckm/pdb). All other data needed to evaluate the conclusions in the paper are present in the paper and/or the Supplementary Information. Solution structure of heparin dp18 is available in the Protein Data Bank with accession number 3IRI (https://doi.org/10.2210/pdb3iri/pdb). The GEO: GSE186216 snRNAseq dataset re-analyzed in this study is available at https://0-www-ncbi-nlm-nih-gov.brum.beds.ac.uk/geo/query/acc.cgi?acc=GSE186216. Source data are provided with this paper.

Research involving human participants, their data, or biological material

Policy information about studies with human participants or human data. See also policy information about sex, gender (identity/presentation), and sexual orientation and race, ethnicity and racism.

Reporting on sex and gender	Not relevant as this study does not involve human participants or respondents, or human data.
Reporting on race, ethnicity, or other socially relevant groupings	Not relevant as this study does not involve human participants or respondents, or human data.
Population characteristics	Not relevant as this study does not involve human participants or respondents, or human data.
Recruitment	Not relevant as this study does not involve human participants or respondents, or human data.
Ethics oversight	Not relevant as this study does not involve human participants or respondents, or human data.

Note that full information on the approval of the study protocol must also be provided in the manuscript.

Field-specific reporting

Please select the one below that is the best fit for your research. If you are not sure, read the appropriate sections before making your selection.

[x] Life sciences [ ] Behavioural & social sciences [ ] Ecological, evolutionary & environmental sciences

For a reference copy of the document with all sections, see nature.com/documents/nr-reporting-summary-flat.pdf

Life sciences study design

All studies must disclose on these points even when the disclosure is negative.

Sample size	Proposed sample sizes were calculated with a detection level of ~25% using G*Power software with α set to 0.05 and the power (1-β) value set to 0.95.
Data exclusions	No data were excluded from the analyses.
Replication	Quantification of GC dendritic spine density was performed independently for n= 3 mice per genotype. Coronal brain sections were analyzed independently for ‘n’ P14 mice for the respective genotypes: WT (n=4), Sema5aGAG/GAG (n=4) , Plxna2+/- (n=5), Plxna2+/-;Sema5a+/- (n=4), Plxna2+/-;Sema5aGAG/+ (n=6) mice.<br>Habenula and fasciculus retroflexus hippocampal regions were analyzed from 3-3 mice with wild-type, Sema5a-/-, and Sema5a GAG/GAG genotypes, respectively.

Randomization | The mice lines used in this study were poor breeders, thus all mice acquired were used in the study, thus randomization was not applicable to this study.

Blinding | The mice lines used in this study were poor breeders, thus all mice acquired were used in the study, thus blinding was not applicable to this study.

Reporting for specific materials, systems and methods

We require information from authors about some types of materials, experimental systems and methods used in many studies. Here, indicate whether each material, system or method listed is relevant to your study. If you are not sure if a list item applies to your research, read the appropriate section before selecting a response.

Materials & experimental systems

n/a	Involved in the study	
☐	☒	Antibodies
☐	☒	Eukaryotic cell lines
☒	☐	Palaeontology and archaeology
☐	☒	Animals and other organisms
☒	☐	Clinical data
☒	☐	Dual use research of concern
☒	☐	Plants

Methods

n/a	Involved in the study	
☒	☐	ChIP-seq
☐	☒	Flow cytometry
☒	☐	MRI-based neuroimaging

Antibodies

Antibodies used | Rat anti-BrdU, 1:500, Abcam ab6326 Lot no: HMBG2351V
chicken anti-GFP, 1:500, Aves Lab #GFP-1020 Lot no: GFP697986
anti-Sema5A rabbit serum diluted 1:500 (in-house produced)

Validation | Rat anti-BrdU antibody
According to the manufacturer's website (https://www.abcam.com/en-hu/products/primary-antibodies/brdu-antibody-bu1-75-icr1-proliferation-marker-ab6326#) this antibody is a proliferation marker that has been validated in IHC-P, Flow Cyt (Intra), ICC/IF and also tested in Chemical samples. Abcam ab6326 has been used in 1397 prior publications.
chicken anti-GFP antibody
According to the manufacturer's website (https://www.aveslabs.com/products/anti-green-fluorescent-protein-antibody-gfp) this antibody is is mixture of IgY fraction and affinity-purified antibodies and validated for ELISA, IHC, ICC, WB applications. Aves Lab #GFP-1020 has been used in 2181 prior publications.
The use of anti-BrdU and anti-GFP for immunofluorescence staining on mouse tissue has been validated and published in (Zhao et al., Cell Reports 2018, (22) 456-470.) by the authors.
The use of anti-Sema5A for Western blot has been validated and published in (Duan et al., Elife, 2014; 3:e04390)

Eukaryotic cell lines

Policy information about cell lines and Sex and Gender in Research

Cell line source(s) | The following cell lines were used in this study:
1.HEK293T cells (ATCC, cat# CRL-3216) commercial cell line
2. CHO GS-/- cells, established in the Clausen laboratory and all described in Chen et al (2018) Nat Methods, PMID: 30104636 and Karlsson et al (2021) Sci. Adv., PMID: 34936441.
These include B4GalT7 KO, Csgalnat1/2/Chsy1 KO, Extl3 KO, Ndst1/2 KO, Hs2st1 KO, Glce KO, Hs6st1-3 KO, NDST2 KI, HS3ST1 KI Csgalnact1/2/Chsy1 KO, HS3ST5 KI Csgalnact1/2/Chsy1 KO, CHST3 KI, CHST15 KI CHO cell lines.

Authentication | None of the cell lines used were authenticated.

Mycoplasma contamination | All cell lines tested negative for mycoplasma contamination.

Commonly misidentified lines (See ICLAC register) | NO commonly misidentified cell lines were used in this study.

Animals and other research organisms

Policy information about studies involving animals; ARRIVE guidelines recommended for reporting animal research, and Sex and Gender in Research

Laboratory animals | Wild type and mutated (Plxna2 and Sema5a) and reporter mice (Thy1-EGFPm) on C57BL/6 background, at ages of (p0, p7, p30 or 3-

	month-old) were used in this study.
Wild animals	This study does not involve wild animals.
Reporting on sex	Male and female mice were included in this study.
Field-collected samples	No field-collected samples are involved in this study.
Ethics oversight	All procedures involving mice were approved by the University of Michigan Institutional Animal Care and Use Committee under protocol PRO00009791 and performed in accordance with guidelines developed by the National Institutes of Health.

Note that full information on the approval of the study protocol must also be provided in the manuscript.

Flow Cytometry

Plots

Confirm that:

- [x] The axis labels state the marker and fluorochrome used (e.g. CD4-FITC).
- [x] The axis scales are clearly visible. Include numbers along axes only for bottom left plot of group (a 'group' is an analysis of identical markers).
- [x] All plots are contour plots with outliers or pseudocolor plots.
- [x] A numerical value for number of cells or percentage (with statistics) is provided.

Methodology

Sample preparation	For each assay sample, 1 × 105 of genetically engineered CHO GS-/- cells were harvested and washed in 1 x PBS before being resuspended in 50 µg/mL of biotinylated WT, R747E/R749E or K734E/R747E/R749E Sema5ATSR3-4 diluted in 1 x PBS with added 1% FBS (assay buffer), gently shaking for 1 h at 4 °C. The cells were then washed with assay buffer before incubation with Alexa Flour 488-streptavidin (1:2,000, #S32354, Invitrogen) diluted in assay buffer while gently shaking for 30 min at 4 ° C. After wash in assay buffer, the cells were resuspended in assay buffer and subjected to flow cytometry on a SA3800 spectral cell analyzer (SONY), where mean fluorescent intensity for each sample was measured. All experiments were performed a minimum of 3 times using triplicate samples, and mean fluorescent intensity was normalized to CHO WT for all samples.
Instrument	SONY SA3800 Spectral cell analyser was used for all binding assays
Software	FlowJo Version 10 was used
Cell population abundance	Not applicable. Gating was performed only to exclude dead cells and doublets.
Gating strategy	The dead cells were excluded based on forward and side scatter area (FSC-A and SSC-A) parameter. Doublets were excluded based on FSC-H (height) and FSC-W (width) parameter.

- [x] Tick this box to confirm that a figure exemplifying the gating strategy is provided in the Supplementary Information.
